# CNGC2 Negatively Regulates Stomatal Closure and Is Not Required for flg22‐ and H_2_O_2_ ‐Induced Guard Cell [Ca^2+^]_cyt_ Elevation in *Arabidopsis thaliana*

**DOI:** 10.1111/ppl.70396

**Published:** 2025-07-09

**Authors:** Rojina Akter, Yasuhiro Inoue, Saori Masumoto, Yoshiharu Mimata, Takakazu Matsuura, Izumi C. Mori, Toshiyuki Nakamura, Yoshimasa Nakamura, Yoshiyuki Murata, Shintaro Munemasa

**Affiliations:** ^1^ Graduate School of Environmental and Life Science Okayama University Okayama Japan; ^2^ Faculty of Agriculture Okayama University Okayama Japan; ^3^ Institute of Plant Science and Resources Okayama University Okayama Japan

**Keywords:** calcium signaling, CNGC, stomata

## Abstract

In guard cells, cytosolic Ca^2+^ acts as a second messenger that mediates abscisic acid (ABA)‐ and pathogen‐associated molecular pattern (PAMP)‐induced stomatal closure. It was reported that Arabidopsis cyclic nucleotide‐gated ion channel 2 (CNGC2) functions as hydrogen peroxide (H_2_O_2_)‐ and PAMP‐activated Ca^2+^‐permeable channels at the plasma membrane of mesophyll cells and mediates Ca^2+^‐dependent PAMP‐triggered immunity. In this study, we examined the role of CNGC2 in the regulation of stomatal movement because *CNGC2* is also expressed in guard cells. We found that stomata of the *CNGC2* disruption mutant *cngc2‐3* are constitutively closed even in the absence of ABA or the flagellar‐derived PAMP, flg22. Consistently, leaf temperatures of the *cngc2‐3* mutant were higher than those of wild‐type (WT) plants. The stomatal phenotype of the *cngc2‐3* mutant was restored by complementation with wild‐type *CNGC2* under the control of the guard cell preferential promoter, *pGC1*. Elevation of cytosolic free Ca^2+^ concentration in guard cells induced by flg22 and H_2_O_2_ remained intact in the *cngc2‐3* mutant. The introduction of the *ost1‐3* mutation into the *cngc2‐3* background did not alter the stomatal phenotype. However, the stomatal phenotype of the *cngc2‐3* mutant was successfully rescued in the double disruption mutant *cngc2‐3aba2‐2.* Taken together, these results suggest that CNGC2 negatively regulates stomatal closure response and does not function as flg22– and H_2_O_2_‐activated Ca^2+^ channels in guard cells. Though CNGC2 is responsive for H_2_O_2_‐ and flg22‐induced [Ca^2+^]_cyt_ elevation in mesophyll cells, the involvement of CNGC2 in the response to H_2_O_2_ and flg22 in guard cells is questionable.

## 1 Introduction

Guard cells can sense various physiological stimuli, including light, drought, CO_2_, and phytohormones such as abscisic acid (ABA) and regulate stomatal apertures, which allow plants to optimize CO_2_ uptake for photosynthesis and transpirational water loss, and adapt to the surrounding environment (Schroeder et al. 2001; Assmann and Jegla 2016). In addition, guard cells can recognize pathogen infection via the pathogen‐associated molecular patterns (PAMPs) and close stomatal pores to prevent pathogen entry into leaves, a process called stomatal immunity (Melotto et al. 2006).

Cytosolic calcium ion (Ca^2+^) in guard cells functions as a second messenger that controls stomatal movement. A rapid increase in cytosolic free Ca^2+^ concentration ([Ca^2+^]_cyt_) in guard cells occurs in response to ABA (McAinsh et al. 1990; Allen et al. 1999), reactive oxygen species (ROS) (Pei et al. 2000), and PAMPs (Thor and Peiter 2014). The guard cell [Ca^2+^]_cyt_ increase activates the plasma membrane anion channel, slow‐anion channel associated 1 (SLAC1) (Mori et al. 2006; Vahisalu et al. 2008; Brandt et al. 2015) and inactivates the plasma membrane (PM) H^+^‐ATPase (Kinoshita et al. 1995), leading to plasma membrane depolarization and thereby stomatal closure. In addition, some other studies have reported the possible involvement of cytosolic Ca^2+^ as a positive regulator for stomatal opening. Blue light triggers guard cell [Ca^2+^]_cyt_ increase via hyperpolarization of guard cell plasma membrane (Harada and Shimazaki 2009). Auxin‐induced stomatal opening is suppressed by the Ca^2+^ chelator, 1,2‐bis(2‐aminophenoxy)ethane‐*N*,*N*,*N′*,*N′*‐tetraacetic acid (BAPTA) in Commelina (Cousson and Vavasseur 1998). Arabidopsis calcineurin B‐like (CBL)‐interacting protein kinase 23 (CIPK23) and CBL complex functions as a [Ca^2+^]_cyt_ sensor that is activated by increased Ca^2+^ concentration (Maierhofer et al. 2014) and positively regulates blue light‐dependent stomatal opening (Inoue et al. 2020). Low CO_2_‐induced stomatal opening is partially mediated by another type of [Ca^2+^]_cyt_ sensor kinases, calcium‐dependent protein kinases (CPKs) (Schulze et al. 2021).

It has been suggested that the major routes for the Ca^2+^ entry in guard cells are plasma membrane Ca^2+^‐permeable ion channels (Hedrich 2012; Jezek and Blatt 2017). Patch‐clamp analyses using Arabidopsis guard cell protoplasts revealed that hyperpolarization‐activated Ca^2+^‐permeable non‐selective cation channels are activated by ABA via NAD(P)H oxidase‐dependent ROS production (Pei et al. 2000; Murata et al. 2001; Kwak et al. 2003). Recently, Tan et al. (2023) reported that the four cyclic nucleotide‐gated channels (CNGCs), CNGC5, 6, 9, and 12, function as ABA‐activated plasma membrane Ca^2+^ channels that are required for ABA‐induced stomatal closure in Arabidopsis. The ABA‐activated protein kinase open stomata 1 (OST1) phosphorylates and activates the CNGCs (Yang et al. 2024), but the CNGCs are not the targets of ROS (Tan et al. 2023), implying that in addition to the CNGCs, molecularly unidentified ROS‐activated Ca^2+^ channels are also involved in Ca^2+^‐dependent ABA signaling. In addition to ABA, PAMPs, including the bacterial flagellar peptide (flg22), bacterial elongation factor (EF‐tu) peptide (elf26), and lipopolysaccharide (LPS) induce stomatal closure via guard cell [Ca^2+^]_cyt_ increase (Arnaud and Hwang 2015; Kim and Liang 2019). It was reported that Arabidopsis reduced hyperosmolality‐induced Ca^2+^ increase 1.3 and 1.6 (OSCA1.3 and 1.6) function as guard cell plasma membrane Ca^2+^ channels that are required for flg22‐induced stomatal closure (Thor et al. 2020). In the double gene disruption mutant *osca1.3osca1.6*, flg22‐induced stomatal closure is impaired, but ABA‐induced stomatal closure remains intact (Thor et al. 2020).

Arabidopsis CNGC2/DND1 was identified from a null mutant ‘defense, no death’ (*dnd1*), which exhibits reduced hypersensitive response (HR) cell death in effector triggered immunity (ETI) (Clough et al. 2000). The CNGC2 together with CNGC4 form a functional Ca^2+^ channel when co‐expressed in *Xenopus* oocytes (Tian et al. 2019). The Arabidopsis *CNGC2*‐defective mutant *cngc2* shows reduction of flg22‐ and hydrogen peroxide (H_2_O_2_)‐triggered calcium channel activation in mesophyll cells (Tian et al. 2019). In addition, the *cngc2* mutant shows reduced flg22‐ and H_2_O_2_‐induced [Ca^2+^]_cyt_ increase, resulting in compromised resistance against pathogen attack. These results suggest that CNGC2 functions as flg22‐ and ROS‐activated Ca^2+^ channels in mesophyll cells and mediates pathogen resistance. *CNGC2* is also highly expressed in guard cells (Wang et al. 2013). A recent study shows that CNGC2 is involved in [Ca^2+^]_cyt_ increase at a whole‐plant level and regulation of stomatal opening induced by high humidity treatment (Hussain et al. 2024). However, the role of CNGC2 in stomatal response to other stimuli and its function in guard cells have not been well established. In this study, we evaluated the role of CNGC2 in the regulation of stomatal movement in response to several stimuli, including flg22 and H_2_O_2_. Our data suggest that CNGC2‐mediated [Ca^2+^]_cyt_ increase functions as a negative regulator of stomatal closure.

## 2 Materials and Methods

### 2.1 Plant Materials and Growth Conditions

*Arabidopsis thaliana* ecotypes Colombia‐0 (Col‐0) and Wassilewskija (Ws) were used as wild‐type (WT) plants. In addition, T‐DNA insertion mutants *cngc2‐3* (Col‐0 background), *cngc2‐2* (Ws background) (Chan et al. 2003), *cngc2‐3ost1‐3* (Col‐0 background), *cngc2‐3aba2‐2* (Col‐0 background), and *CNGC2* complementation line (*cngc2‐3pGC1::CNGC2*) were used for this study. The *CNGC2* and *OST1* loss‐of‐function mutant *cngc2‐3ost1‐3* was generated by crossing T‐DNA insertion mutants *cngc2‐3* (SALK_066908) (Chin et al. 2013) and *ost1‐3* (SALK_008068) (Yoshida et al. 2002). Similarly, the double disruption mutant *cngc2‐3aba2‐2* was also generated by crossing between the T‐DNA insertion mutant *cngc2‐3* and fast neutron‐generated mutant *aba2‐2* (Nambara et al. 1998). For the generation of the complemented line in the background of *cngc2‐3*, the guard cell preferential promoter *pGC1* (Yang et al. 2008), wild‐type *CNGC2* coding sequence, and Venus were cloned into the pHygII‐UT vector (Waadt et al. 2008; Kunz et al. 2014). *Agrobacterium tumefaciens* GV3101 carrying the construct was used to transform the *cngc2‐3* mutant by the floral dip method (Clough and Bent 1998). All these Arabidopsis seeds were sown on a soil mixture of 1:1 soil: vermiculite (v/v) and grown in growth chambers with controlled conditions (16/8 h light/dark condition at 22°C temperature, 80 μmol m^−2^ s^−1^ light intensity, and 70% relative humidity) after being stratified at 4°C for 3 days. Plants were watered twice a week with 0.1% (v/v) Hyponex. For all experiments, rosette leaves from 3‐ to 5‐week‐old plants were used.

### 2.2 Measurement of Stomatal Aperture

Stomatal apertures were measured as described previously (Munemasa et al. 2019) with a few modifications. Fully expanded rosette leaves were detached and floated on stomatal bioassay buffer containing 5 mM KCl, 50 μM CaCl_2_, and 10 mM MES/Tris (pH 5.6). The leaves were incubated for 2 h in light to induce stomatal opening and then treated with 10 μM ABA for 2 h or 2 μM flg22 for 1 h in the stomatal assay buffer. To determine the stomatal opening by fusicoccin (FC), leaves were incubated in stomatal opening buffer for 2 h in the dark. Then 10 μM FC was added and incubated in the dark for 2 h. After incubation, the treated leaves were shredded in a blender, and the epidermal peels were collected with a nylon mesh. The peels were placed on a glass slide and photographed under an optical microscope (IX71, Olympus). At least 20 stomatal apertures of a leaf of one plant were measured per independent experiment using WinRoof3.0 software (Mitani Corporation) to calculate an average. The experiments were performed with at least three independent replications using different plants to obtain the average (*n* = 3, 60 stomata).

### 2.3 Determination of Leaf Temperature

For leaf temperature measurement, 3‐ to 4‐week‐old plants grown in the soil were considered. Leaf temperature was measured using an infrared thermography instrument (InfReC R550/R450; Nippon Avionics Co. Ltd.) and analyzed images using the InfReC Analyzer NS9500 standard software.

### 2.4 Cloning and cRNA Synthesis for Two‐Electrode Voltage‐Clamp Analysis

*CNGC2* (AT5G15410), *CNGC4* (AT5G54250), and *AtCaM4* (AT1G66410) were cloned into the pNB1u oocyte expression vector by the USER cloning strategy (Nour‐Eldin et al. 2006). The capped RNA (cRNA) was synthesized from 1 μg of linearized plasmid DNA template using the mMESSAGE mMACHINE T7 Transcription kit (Thermo Fisher Scientific), according to the manufacturer's instructions. The quality of synthesized cRNA was checked by agarose gel electrophoresis.

### 2.5 Two‐Electrode Voltage‐Clamp Analysis

Each *Xenopus laevis* oocyte was injected with 50 mL of water or cRNA (each 10 ng) and incubated in ND96 buffer (96 mM NaCl, 2 mM KCl, 1 mM MgCl_2_, 1 mM CaCl_2_, 10 mM HEPES/NaOH, pH 7.5) at 18°C for a few days before electrophysiological recordings previously described by Tian et al. (2019). The glass pipettes were filled with 3 M KCl. The bath solution contained 5 mM CaCl_2_, 2 mM KCl, 1 mM MgCl_2_, and 10 mM MES/Tris to adjust the pH to 5.6. The osmolality was adjusted to 220 mM using D‐mannitol. The oocytes were continuously perfused with the bath solution during the experiment. Currents were recorded with a holding potential of 0 mV and ranging from +40 to −180 mV in 20‐mV decrements. Voltage‐clamp recordings were performed using an Axoclamp 900A amplifier (Molecular Devices); data were recorded using a Digidata 1440A system (Molecular Devices) and analyzed using PCLAMP 10.7 software (Molecular Devices).

### 2.6 Guard Cell [Ca^2+^]_cyt_ Imaging

Transgenic Arabidopsis plants expressing Nuclear Export Signal (NES)‐fused Yellow Cameleon3.6 (NES‐YC3.6) under the control of the guard cell preferential promoter *pGC1* were used in this study (Yang et al. 2008; Krebs et al. 2012). The rosette leaves of 3‐ to 4‐week‐old seedlings were detached, and the abaxial side of the leaf was gently adhered to a cover glass with an adhesive. Then, abaxial epidermal strips of leaves were peeled off, and mesophyll tissues were removed with a sharp razor blade. The epidermal strips were immersed in stomatal opening buffer and incubated under light for at least 2 h at 22°C. Turgid guard cells were selected and used for [Ca^2+^]_cyt_ imaging. 5 min after starting the experiment, guard cells were treated with 500 nM flg22, 500 μM H_2_O_2_, 50 μM ABA, or 10 mM CaCl_2_, and the response was monitored for 30 min. Fluorescence intensities (F535 and F480) from the cyan fluorescent protein (CFP) and yellow fluorescent protein (YFP) in guard cells were measured under a fluorescence microscope (IX73, Olympus) equipped with a dual‐emission imaging system (W‐View Gemini system; 440AF21 excitation filter, 445DRLP dichroic mirror and 2 emission filters, 480DF30 for CFP and 535DF25 for YFP, Hamamatsu Photonics) and a CMOS camera (Hamamatsu ORCA‐Flash4.0 digital camera, Hamamatsu Photonics). The same exposure time was used for both CFP and YFP and for the analysis. [Ca^2+^]_cyt_ elevation was counted when changes in fluorescence ratios (F535/F480) were ≥ 0.1 unit from the baseline.

### 2.7 ABA Content Measurement

Approximately 0.1 g (fresh weight) of rosettes were frozen in liquid nitrogen, ground, and extracted with 4 mL of extraction solvent (80% acetonitrile, 19% water, 1% acetic acid) containing isotope‐labelled internal standards (d_6_‐ABA; OlChemim s.r.o.). After incubation for 1 h at 4°C and centrifugation, the supernatant was combined with a rinse of the pellet and purified by sequential solid‐phase extraction using Oasis HLB, MCX, and WAX cartridges (Waters Corporation), as described in Gupta et al. (2017). The final ABA fraction, eluted with extraction solvent from the WAX cartridge, was dried and reconstituted in 100 μL of 1% aqueous acetic acid. ABA levels were quantified using an Agilent 1260–6410 Triple Quadrupole LC–MS system equipped with a ZORBAX Eclipse XDB‐C18 column. Chromatographic conditions followed Tsukahara et al. (2015), using a solvent gradient (3%–50% acetonitrile with acetic acid) over 20 min at a flow rate of 4 mL min^−1^. MS/MS transitions were m/z 263/153 for ABA and m/z 269/159 for d_6_‐ABA, in negative ion mode, with a retention time of approximately 12.6 min.

### 2.8 Statistical Analysis

For data analysis, the chi‐squared (*χ*2) test and one‐way ANOVA with Tukey's test were used to assess significant differences between datasets. For significant differences, *p* values < 0.05 were considered.

## 3 Results

### 3.1 Disruption of CNGC2 Confers Constitutive Stomatal Closing Phenotype in Arabidopsis

**FIGURE 1:**
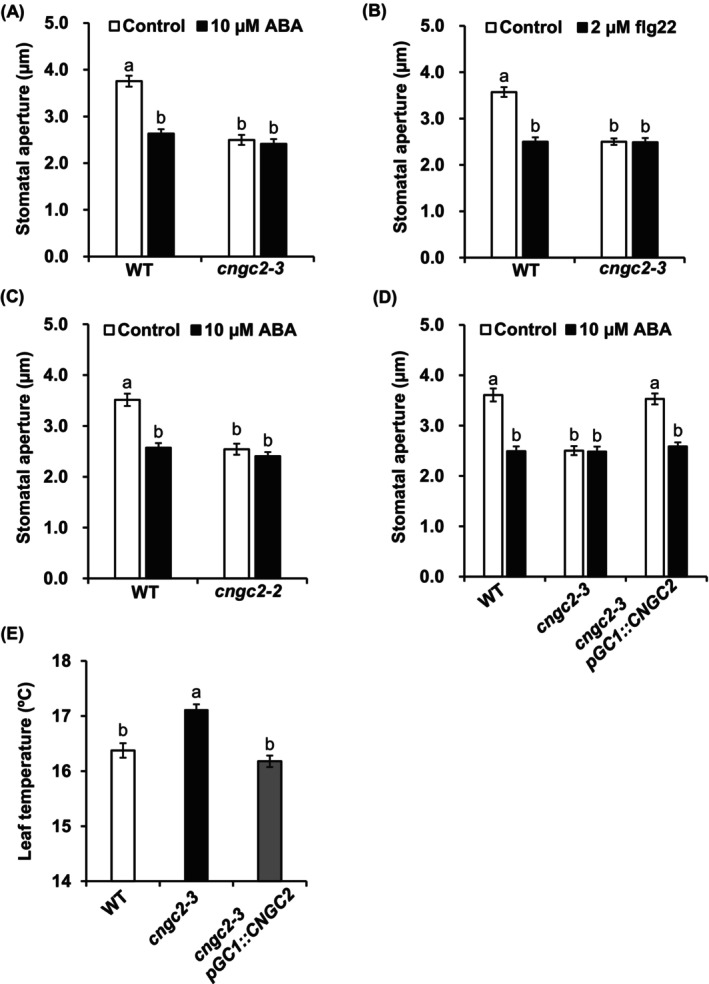
Stomatal phenotype of the *cngc2‐3* mutant. (A) 10 μM ABA‐induced stomatal closure in Columbia‐0 (Col‐0) as wild‐type (WT) and *cngc2‐3* (Col‐0 background) plants. (B) 2 μM flg22‐triggered stomatal closure in WT (Col‐0) and *cngc2‐3* (Col‐0 background) plants. (C) 10 μM ABA‐induced stomatal closure in Wassilewskija (Ws) as wild‐type (WT) and *cngc2‐2* (Ws background) plants. (D) 10 μM ABA‐induced stomatal closure in WT (Col‐0), *cngc2‐3* (Col‐0 background), and *CNGC2* complemented line (*cngc2‐3pGC1::CNGC2*). (A–D) Averages of stomatal apertures from three independent experiments (*n* = 3, total 60 stomata) are shown. (E) Leaf temperature of WT (Col‐0), *cngc2‐3* (Col‐0 background), and *cngc2‐3pGC1::CNGC2 plants* under normal growth conditions. The temperature of nine leaves was determined in triplicate. Data are expressed as mean ± SE. Statistical differences were analyzed by one‐way ANOVA with Tukey's test. Different letters represent significant differences (*p* < 0.05).

To evaluate functions of the Arabidopsis plasma membrane Ca^2+^‐permeable channel CNGC2 in stomatal movement, first, we examined the stomatal response of the *cngc2‐3* mutant (Col‐0 background) and *cngc2‐2* mutant (Ws background). The experiment was carried out with detached leaves. Application of ABA at 10 μM or flg22 at 2 μM induced stomatal closure in wild‐type plants, but the stomatal pores of the *cngc2‐3* mutant were closed even without ABA or flg22 treatment (Figure 1A,B). A similar phenotype was also observed in the *cngc2‐2* mutant (Figure 1C). Complementation of the *cngc2‐3* mutation with the wild‐type CNGC2 fused with Venus under control of guard cell preferential promoter *pGC1* (Yang et al. 2008) restored the stomatal response (Figure 1D), indicating that disruption of *CNGC2* expressed in guard cells is responsible for the *cngc2‐3* phenotype. Consistent with the result of stomatal aperture measurement, the leaf temperature of the *cngc2‐3* mutant was higher than that of wild‐type plants, and there was no significant difference in leaf temperature between wild‐type plants and the guard cell *CNGC2* complementation line (*cngc2‐3pGC1::CNGC2*) (Figure 1E). To check if *CNGC2* disruption causes a complete loss of stomatal opening ability, we performed stomatal aperture measurement with a fungal phytotoxin fusicoccin (FC) that causes stomatal opening through activation of the plasma membrane H^+^‐ATPase in guard cells (Kinoshita and Shimazaki 2001). Similar to wild‐type, the *cngc2‐3* mutant opened stomata in response to FC treatment (Figure S1), indicating that the *cngc2‐3* mutant is defective in guard cell signaling that regulates stomatal movement, but not in the development of functional stomata.

### 3.2 CNGC2 Is Not Involved in flg22‐ and H_2_O_2_ ‐Induced [Ca^2+^]_cyt_ Elevation in Guard Cells

**FIGURE 2:**
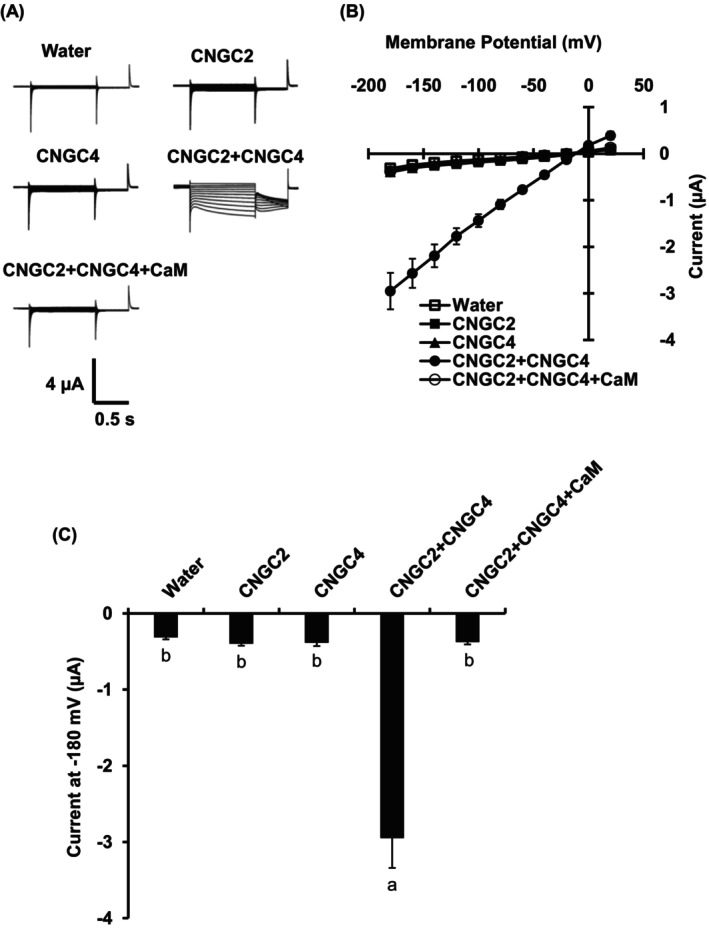
CNGC2 together with CNGC4 forms a functional Ca^2+^ channel in *Xenopus* oocytes. (A) Two‐electrode voltage‐clamp recording from *Xenopus* oocytes expressing various CNGCs. Currents were recorded with a holding potential of 0 mV and ranging from +40 to −180 mV in 20 mV decrements. (B) Average steady‐state current–voltage curves of whole‐cell negative current recordings. (C) Current amplitudes at −180 mV. Data are the mean ± SE (*n* = 6, independent oocytes). Different letters indicate significant differences at *p* < 0.05.

In vitro two‐electrode voltage clamp analysis revealed that CNGC2, together with CNGC4, forms a functional Ca^2+^ channel in *Xenopus* oocytes (Figure 2; Tian et al. 2019). In mesophyll cells, CNGC2 is required for H_2_O_2_‐ and PAMP‐induced [Ca^2+^]_cyt_ elevation (Tian et al. 2019). Next, we tested whether CNGC2 is also involved in PAMP‐ and H_2_O_2_‐induced [Ca^2+^]_cyt_ elevation in guard cells using the genetically encoded [Ca^2+^]_cyt_ indicator, NES‐YC3.6. We monitored [Ca^2+^]_cyt_ elevation in guard cells using isolated epidermal strips.

**FIGURE 3:**
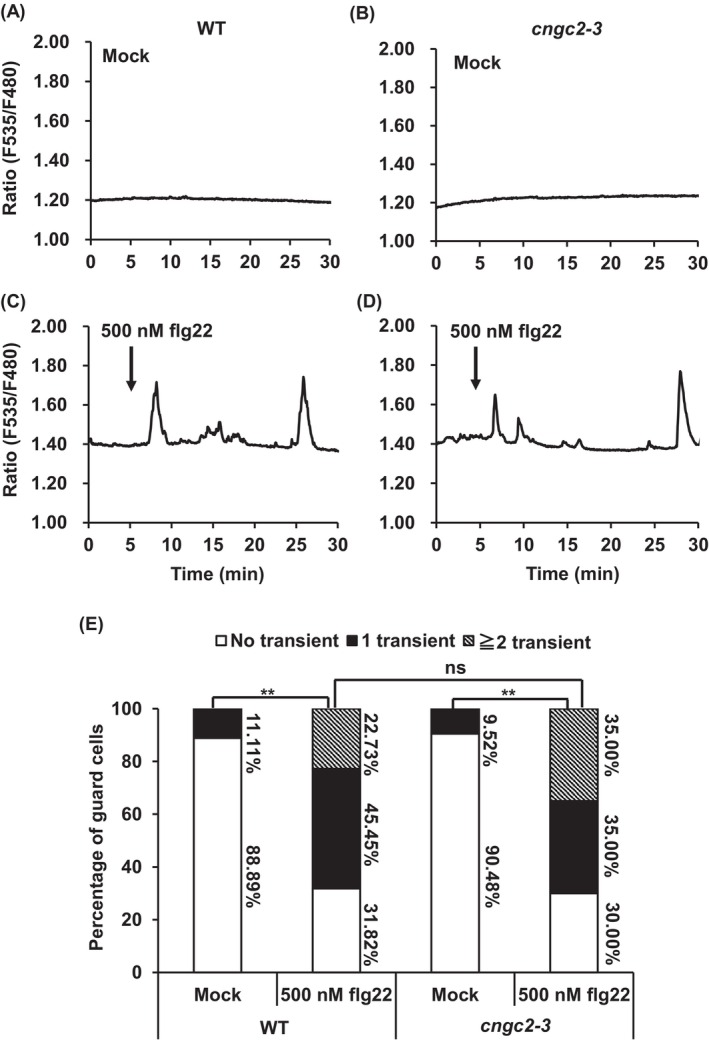
The *cngc2‐3* mutant showed flg22‐induced cytosolic calcium ([Ca^2+^]_cyt_) elevation in guard cells. The guard cell [Ca^2+^]_cyt_ elevation of wild‐type (WT) and *cngc2‐3* plants expressing Nuclear Export Signal (NES)‐fused Yellow Cameleon3.6 (NES‐YC3.6) was monitored. (A–D) Representative traces of fluorescence emission ratios (F535/F480) showing flg22‐induced [Ca^2+^]_cyt_ transients in guard cells. In mock treatments, guard cells were incubated in stomatal assay buffer for 2 h in light, and no flg22 applied during the measurement of fluorescence emission ratios (A, B). In flg22 treatments, 500 nM flg22 were applied to the guard cells in stomatal assay buffer 5 min after the measurement (C, D). (E) Percentage bar chart represents the percent (%) of guard cells showing transient [Ca^2+^]_cyt_ elevations. The number of transient [Ca^2+^]_cyt_ elevations under mock and 500 nM flg22 treatment in WT and *cngc2‐3* mutants were counted when the fluorescence ratio (F535/F480) increased ≥ 0.1 unit from the baseline. In this figure, Col‐0 was used as wild‐type (WT). The significance of differences between different treatments were determined by chi‐squared (*χ*2) test, *p < 0.05, **p < 0.01. The “ns” indicates non‐significant difference where *p* > 0.05.

**FIGURE 4:**
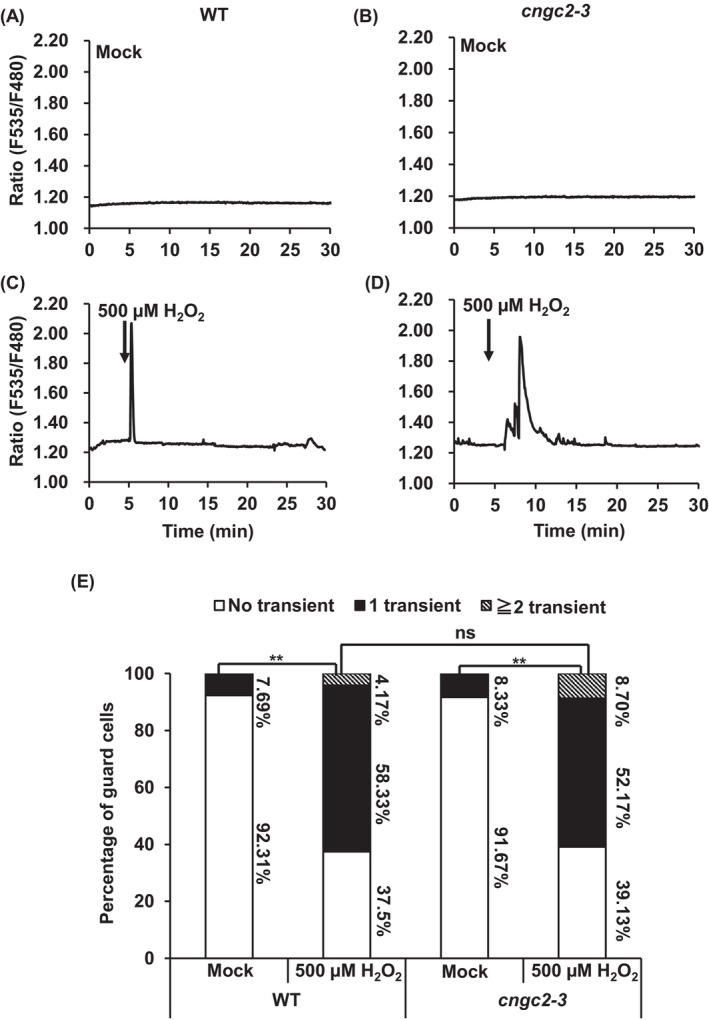
The *cngc2‐3* mutant showed H_2_O_2_‐induced cytosolic calcium ([Ca^2+^]_cyt_) elevation in guard cells. The guard cell [Ca^2+^]_cyt_ elevation of wild‐type (WT) and *cngc2‐3* plants expressing Nuclear Export Signal (NES)‐fused Yellow Cameleon3.6 (NES‐YC3.6) was monitored. (A–D) Representative traces of fluorescence emission ratios (F535/F480) showing H_2_O_2_‐induced [Ca^2+^]_cyt_ transients in guard cells. In mock treatments, guard cells were incubated in stomatal assay buffer for 2 h in light, and no H_2_O_2_ applied during the measurement of fluorescence emission ratios (A, B). In H_2_O_2_ treatments, 500 μM H_2_O_2_ were applied to the guard cells in stomatal assay buffer 5 min after the measurement (C, D). (E) Percentage bar chart represents the percent (%) of guard cells showing transient [Ca^2+^]_cyt_ elevations. The number of transient [Ca^2+^]_cyt_ elevations under mock and 500 μM H_2_O_2_ treatment in WT and *cngc2‐3* mutants were counted when the fluorescence ratio (F535/F480) increased ≥ 0.1 unit from the baseline. In this figure, Col‐0 was used as wild‐type (WT). The significance of differences between different treatments were determined by chi‐squared (*χ*2) test, *p < 0.05, **p < 0.01. The “ns” indicates non‐significant difference where *p* > 0.05.

The [Ca^2+^]_cyt_ spikes (transient [Ca^2+^]_cyt_ elevations) were observed in 11.11% of wild‐type guard cells treated without flg22 (*n* = 18) (Figure 3A,E) and 68.18% of guard cells treated with 500 nM flg22 (*n* = 22) (Figure 3C,E). The frequency of [Ca^2+^]_cyt_ spikes in wild‐type guard cells treated with 500 nM flg22 was significantly larger than that in the wild‐type guard cells treated without flg22 (*p* < 0.01) (Figure 3E), as previously reported (Thor and Peiter 2014). In the *cngc2‐3* mutant, Ca^2+^ peaks were found in 9.52% of guard cells treated with no flg22 (*n* = 21) (Figure 3B,E) and 70.00% of guard cells treated with 500 nM flg22 (*n* = 20) (Figure 3D,E). The number of [Ca^2+^]_cyt_ spikes in the guard cells of *cngc2‐3* treated with 500 nM flg22 was significantly higher than that in the *cngc2‐3* guard cells treated without flg22 (*p* < 0.01) (Figure 3E). The percentage of guard cells that showed [Ca^2+^]_cyt_ spikes in the *cngc2‐3* guard cells treated with 500 nM flg22 was not significantly different from those in wild‐type guard cells treated with 500 nM flg22 (Figure 3E). Next, we evaluated the H_2_O_2_‐induced [Ca^2+^]_cyt_ elevation in guard cells. Similar to flg22, 500 μM H_2_O_2_ treatment increased percentages of guard cells showing [Ca^2+^]_cyt_ spikes in both wild‐type and *cngc2‐3* guard cells, and there was no significant difference between wild‐type and *cngc2‐3* guard cells showing the H_2_O_2_‐induced [Ca^2+^]_cyt_ spikes (Figure 4E). We also tested whether *CNGC2* is involved in guard cells [Ca^2+^]_cyt_ increases induced by other stimuli, including ABA and high extracellular Ca^2+^. We found that treatment with 50 μM ABA or 10 mM CaCl_2_ induced [Ca^2+^]_cyt_ elevations in both wild‐type and *cngc2‐3* guard cells (Figures S2 and S3) and no significant difference was observed in ABA‐ and high extracellular Ca^2+^‐triggered guard cells [Ca^2+^]_cyt_ elevation between wild‐type and *cngc2‐3* mutant (Figures S2 and S3). A well‐known plasma membrane channel blocker, lanthanum chloride (LaCl_3_) completely inhibited the flg22‐, H_2_O_2_‐, ABA‐, and high extracellular Ca^2+^‐induced [Ca^2+^]_cyt_ spikes (Data not shown), indicating that all the stimuli trigger [Ca^2+^]_cyt_ elevation through the activation of the plasma membrane Ca^2+^ channels, as previously reported (Pei et al. 2000; Murata et al. 2001; Thor et al. 2020). Taken together, these results suggest that CNGC2 is not required in flg22‐, H_2_O_2_‐, ABA‐, and high extracellular Ca^2+^‐induced [Ca^2+^]_cyt_ increase in guard cells.

### 3.3 The *cngc2‐3* Phenotype Is Dependent on Endogenous ABA but Not OST1 Kinase

**FIGURE 5:**
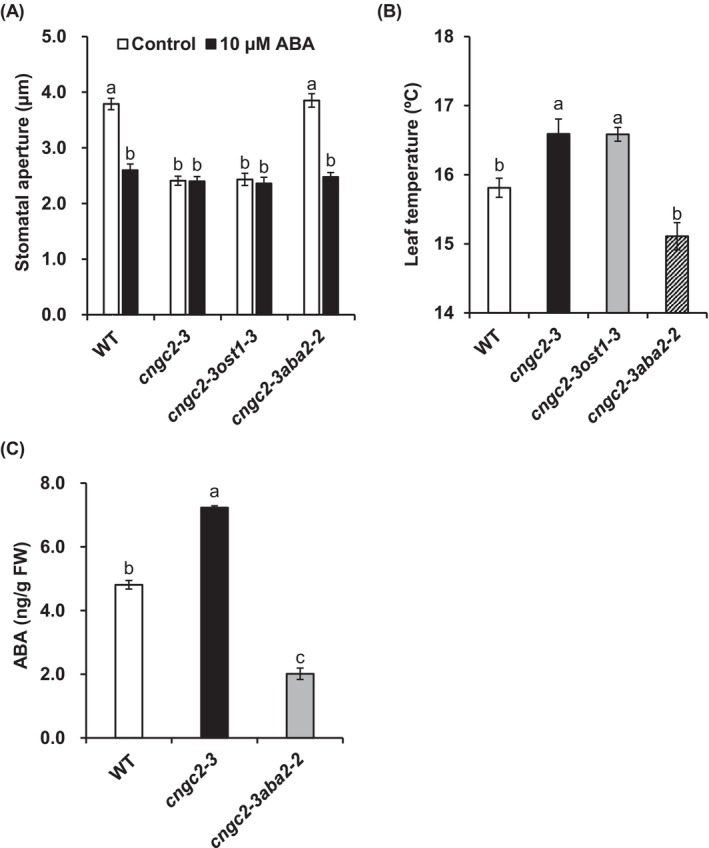
Stomatal phenotype of the *cngc2‐3* mutant is dependent on endogenous ABA but not on OST1 kinase. (A) 10 μM ABA‐induced stomatal closure in wild‐type (WT), *cngc2‐3*, *cngc2‐3ost1‐3*, and *cngc2‐3aba2‐2* plants. Averages of stomatal apertures from three independent experiments (*n* = 3, total 60 stomata) are shown. (B) Leaf temperature of WT, *cngc2‐3, cngc2‐3ost1‐3*, and *cngc2‐3aba2‐2* plants under normal growth conditions. The temperature of nine leaves was measured in triplicate. (C) ABA accumulation in WT, *cngc2‐3, and cngc2‐3aba2‐2* plants. Col‐0 was used as wild‐type (WT) for all experiments in this figure. Values represent mean ± SE. Statistical differences were analyzed by one‐way ANOVA with Tukey's test. Different letters represent significant differences (*p* < 0.05).

To analyze the mechanism that causes the *cngc2‐3* stomatal phenotype, we performed stomatal aperture measurements using the double disruption mutants *cngc2‐3ost1‐3* and *cngc2‐3aba2‐2*. The Ca^2+^‐independent protein kinase OST1 is one of the major components for Ca^2+^‐independent ABA signaling in guard cells (Mustilli et al. 2002) and phosphorylates the downstream targets, including SLAC1 (Geiger et al. 2009; Lee et al. 2009) and CNGC5, 6, 9, and 12 (Yang et al. 2024). The short‐chain alcohol dehydrogenase ABA2 converts xanthoxin to abscisic aldehyde, and *ABA2* disruption causes a severe ABA‐deficient phenotype (Cheng et al. 2002; González‐Guzmán et al. 2002). We found that, similar to the *cngc2‐3* mutant, the *cngc2‐3ost1‐3* mutant showed a constitutive closed stomatal phenotype (Figure 5A). However, unlike the *cngc2‐3* mutant and *cngc2‐3ost1‐3* double mutant, the *cngc2‐3aba2‐2* double mutant showed a wild‐type‐like stomatal phenotype (Figure 5A), indicating that the introduction of the *aba2‐2* mutation into the *cngc2‐3* background restored the stomatal phenotype. Consistently, the *cngc2‐3ost1‐3* double mutant showed higher leaf temperature compared to the wild‐type plant, but no significant difference was observed in leaf temperature between the wild‐type and *cngc2‐3aba2‐2* double mutant (Figure 5B). Next, to check whether the constitutive closed stomatal phenotype of the *cngc2‐3* mutant is due to ABA over‐accumulation, we measured the ABA content in rosette leaves. We found that the ABA content in *cngc2‐3* rosette leaves was significantly, but only 1.5 times higher than that in wild‐type rosette leaves (Figure 5C).

## 4 Discussion

Cytosolic Ca^2+^ plays a key role in regulating guard cell ion transport and stomatal movements (McAinsh et al. 1990; Mori et al. 2006; Siegel et al. 2009; Chen et al. 2010; Wang et al. 2013). The role of Ca^2+^ as a positive regulator in stomatal closure has been well established (De Silva et al. 1985; Pei et al. 2000; Hossain et al. 2011; Murata et al. 2015; Agurla et al. 2018; Tian et al. 2019). The involvement of Ca^2+^ as a positive regulator in stomatal opening has also been suggested in some studies (Shimazaki et al. 1992; Cousson and Vavasseur 1998; Harada and Shimazaki 2009; Inoue et al. 2020; Schulze et al. 2021). In guard cells, the plasma membrane Ca^2+^ channels act as the core players that trigger the stimuli‐induced [Ca^2+^]_cyt_ elevation. The Arabidopsis plasma membrane Ca^2+^ channel CNGC2 is involved in various physiological processes including pathogen resistance (Yu et al. 1998; Clough et al. 2000; Tian et al. 2019), calcium sensitivity (Chan et al. 2003; Wang et al. 2017), flower development (Chaiwongsar et al. 2009; Chin et al. 2013), heat tolerance (Finka et al. 2012; Lu et al. 2022), and auxin homeostasis (Chakraborty et al. 2021). Recently, Tian et al. (2019) reported that in mesophyll cells, CNGC2 functions as a plasma membrane Ca^2+^‐permeable channel that mediates H_2_O_2_ and flg22 responses. CNGC2 is also expressed in guard cells (Wang et al. 2013) and guard cells close stomata in response to H_2_O_2_ (Pei et al. 2000) and flg22 (Melotto et al. 2006). Therefore, here we analyzed the role of CNGC2 in the regulation of guard cell signaling and stomatal movement.

In this study, we found that the *cngc2* mutants showed constantly closed stomata (Figure 1A–C), as recently reported (Hussain et al. 2024). In addition, the complementation analysis revealed that disruption of guard cell‐expressed *CNGC2* is responsible for the *cngc2* stomatal phenotype (Figure 1D). Consistently, the *cngc2‐3* mutant showed higher leaf temperature than that of wild‐type and *CNGC2* complementation line under control growth conditions (Figure 1E). These results suggest that guard cell CNGC2 is a negative regulator of stomatal closure response.

Although CNGC2 forms a functional Ca^2+^‐permeable cation channel (Figure 2) and is responsive for H_2_O_2_‐ and flg22‐induced [Ca^2+^]_cyt_ elevation and immune responses in mesophyll cells (Tian et al. 2019), flg22‐ and H_2_O_2_‐induced [Ca^2+^]_cyt_ elevation remained intact in the *cngc2‐3* guard cells (Figures 3 and 4). Therefore, the function of CNGC2 as a flg22‐ and H_2_O_2_‐activated Ca^2+^ permeable cation channel might be cell‐type specific. In addition to flg22 and H_2_O_2_, ABA and high extracellular Ca^2+^, stimuli that induce stomatal closure, were able to induce guard cell [Ca^2+^]_cyt_ elevation in the *cngc2‐3* mutant as in wild‐type plants (Figures S2 and S3). This result suggests that CNGC2 does not function as Ca^2+^ permeable channels responsible for stomatal closure induced by ABA and high extracellular Ca^2+^.

The introduction of the *aba2‐2* mutation into the *cngc2‐3* background restored the stomatal phenotype of the *cngc2‐3* mutant (Figure 5A). On the other hand, the double disruption mutant *cngc2‐3ost1‐3* showed the constitutively closed stomatal phenotype as the *cngc2‐3* mutant did (Figure 5A). These results suggest that the stomatal phenotype of the *cngc2‐3* mutant is dependent on endogenous ABA, but not the OST1 kinase. Overaccumulation of ABA may be the possible cause of the constitutive closed stomatal phenotype of the *cngc2‐3* mutant (Figure 5C). However, since it has been reported that ABA contents in Arabidopsis normally increase 10‐ to 100‐fold in response to drought stress and other conditions (Kale et al. 2019; Sato et al. 2018; Gupta et al. 2017), solid conclusions cannot be drawn from the slight increase (1.5‐fold) in ABA content observed in the *cngc2‐3* mutant.

A recent study reported that CNGC2 mediates high humidity‐induced stomatal opening (Hussain et al. 2024). *CYP707A3*, encoding ABA 8′‐hydroxylase, is responsible for the inactivation of the major ABA pool under high humidity conditions (Okamoto et al. 2009). High humidity triggers CNGC2‐dependent [Ca^2+^]_cyt_ increase at the outermost regions of leaves, which leads to the activation of calmodulin (CaM)‐binding transcriptional activators (CAMTAs), resulting in the induction of *CYP707A3* expression. The CNGC2/CAMTA‐dependent transcriptional activation of *CYP707A3* causes ABA catabolism and subsequent stomatal opening (Hussain et al. 2024). This is consistent with our data showing a slight increase in ABA contents in the *cngc2‐3* mutant grown under the condition where the relative humidity is 70% (Figure 5C). We found that the disruption of the guard cell core ABA signaling component OST1 kinase did not restore the stomatal phenotype of the *cngc2‐3* mutant (Figure 5A), suggesting that, unlike low humidity‐induced stomatal closure (Merilo et al. 2013; Merilo et al. 2018), high humidity‐induced stomatal opening is less dependent on the OST1 kinase. This could be explained by high humidity‐activated ABA‐dependent but OST1‐independent signaling, but the details need to be studied in the future.

In conclusion, our findings suggest that, unlike mesophyll cells, guard cells do not utilize CNGC2 as a Ca^2+^‐permeable channel that is required for flg22‐ and H_2_O_2_‐induced [Ca^2+^]_cyt_ increase. CNGC2 functions as a negative regulator of stomatal closure through ABA‐dependent, but OST1‐independent pathways.

## Author Contributions

**Rojina Akter:** conceptualization, investigation, writing – original draft, writing – review and editing. **Yasuhiro Inoue:** methodology. **Saori Masumoto:** methodology. **Yoshiharu Mimata:** methodology. **Takakazu Matsuura:** investigation. **Izumi C. Mori:** investigation, writing – original draft. **Toshiyuki Nakamura:** supervision. **Yoshimasa Nakamura:** supervision. **Yoshiyuki Murata:** supervision, funding acquisition, writing – review and editing. **Shintaro Munemasa:** conceptualization, investigation, supervision, funding acquisition, writing – original draft, writing – review and editing.

## Conflicts of Interest

The authors declare no conflicts of interest.

## Supporting information


**Figure S1.** Fusicoccin (FC)‐mediated stomatal opening in WT and the *cngc2‐3* mutant. Averages of stomatal apertures from three independent experiments (*n* = 3, total 60 stomata) are shown. In this figure, Col‐0 was used as wild‐type (WT). Data are expressed as mean ± SE. Statistical differences were analyzed by one‐way ANOVA with Tukey’s test. Different letters represent significant differences (*p* < 0.05).
**Figure S2.** The *cngc2‐3* mutant showed ABA‐induced cytosolic calcium ([Ca^2+^]_cyt_) elevation in guard cells. The guard cell [Ca^2+^]_cyt_ elevation of wild‐type (WT) and *cngc2‐3* plants expressing Nuclear Export Signal (NES)‐fused Yellow Cameleon3.6 (NES‐YC3.6) was monitored. (A‐D) Representative traces of fluorescence emission ratios (F535/F480) showing ABA‐induced [Ca^2+^]_cyt_ transients in guard cells. In mock treatments, guard cells were incubated in stomatal assay buffer for 2 h in light, and no ABA applied during the measurement of fluorescence emission ratios (A, B). In ABA treatments, 50 μM ABA were applied to the guard cells in stomatal assay buffer 5 min after the measurement (C, D). (E) Percentage bar chart represents the percent (%) of guard cells showing transient [Ca^2+^]_cyt_ elevations. The number of transient [Ca^2+^]_cyt_ elevations under mock and 50 μM ABA treatment in WT and *cngc2‐3* mutants were counted when the fluorescence ratio (F535/F480) increased ≥ 0.1 unit from the baseline. In this figure, Col‐0 was used as wild‐type (WT). The significance of differences between different treatments were determined by chi‐squared (*χ*2) test, *p < 0.05, **p < 0.01. The “ns” indicates non‐significant difference where *p* > 0.05.
**Figure S3.** The *cngc2‐3* mutant showed high extracellular Ca^2+^‐induced cytosolic calcium ([Ca^2+^]_cyt_) elevation in guard cells. The guard cell [Ca^2+^]_cyt_ elevation of wild‐type (WT) and *cngc2‐3* plants expressing Nuclear Export Signal (NES)‐fused Yellow Cameleon3.6 (NES‐YC3.6) was monitored. (A–D) Representative traces of fluorescence emission ratios (F535/F480) showing extracellular Ca^2+^‐induced [Ca^2+^]_cyt_ transients in guard cells. In mock treatments, guard cells were incubated in stomatal assay buffer for 2 h in light, and no extracellular Ca^2+^ applied during the measurement of fluorescence emission ratios (A, B). In extracellular Ca^2+^ treatments, 10 mM CaCl_2_ were applied to the guard cells in stomatal assay buffer 5 min after the measurement (C, D). (E) Percentage bar chart represents the percent (%) of guard cells showing transient [Ca^2+^]_cyt_ elevations. The number of transient [Ca^2+^]_cyt_ elevations under mock and 10 mM CaCl_2_ treatment in WT and *cngc2‐3* mutants were counted when the fluorescence ratio (F535/F480) increased ≥ 0.1 unit from the baseline. In this figure, Col‐0 was used as wild‐type (WT). The significance of differences between different treatments were determined by chi‐squared (*χ*2) test, *p < 0.05, **p < 0.01. The “ns” indicates non‐significant difference where *p* > 0.05.

## Data Availability

The data will be available on request.
