# Supplementary material for: CNGC2 Negatively Regulates Stomatal Closure and Is Not Required for flg22‐ and H2O2 ‐Induced Guard Cell [Ca2+]cyt Elevation in Arabidopsis thaliana
Source: Physiol Plant. 2025 Jul 9;177(4):e70396. doi: 10.1111/ppl.70396 (PMC12241493; doi:10.1111/ppl.70396)
Supplement: Supplementary file 1 — Figure S1. Fusicoccin (FC)‐mediated stomatal opening in WT and the cngc2‐3 mutant. Averages of stomatal apertures from three independent experiments (n = 3, total 60 stomata) are shown. In this figure, Col‐0 was used as wild‐type (WT). Data are expressed as mean ± SE. Statistical differences were analyzed by one‐way ANOVA with Tukey’s test. Different letters represent significant differences (p < 0.05). Figure S2. The cngc2‐3 mutant showed ABA‐induced cytosolic calcium ([Ca2+]cyt) elevation in guard cells. The guard cell [Ca2+]cyt elevation of wild‐type (WT) and cngc2‐3 plants expressing Nuclear Export Signal (NES)‐fused Yellow Cameleon3.6 (NES‐YC3.6) was monitored. (A‐D) Representative traces of fluorescence emission ratios (F535/F480) showing ABA‐induced [Ca2+]cyt transients in guard cells. In mock treatments, guard cells were incubated in stomatal assay buffer for 2 h in light, and no ABA applied during the measurement of fluorescence emission ratios (A, B). In ABA treatments, 50 μM ABA were applied to the guard cells in stomatal assay buffer 5 min after the measurement (C, D). (E) Percentage bar chart represents the percent (%) of guard cells showing transient [Ca2+]cyt elevations. The number of transient [Ca2+]cyt elevations under mock and 50 μM ABA treatment in WT and cngc2‐3 mutants were counted when the fluorescence ratio (F535/F480) increased ≥ 0.1 unit from the baseline. In this figure, Col‐0 was used as wild‐type (WT). The significance of differences between different treatments were determined by chi‐squared (χ2) test, *p < 0.05, **p < 0.01. The “ns” indicates non‐significant difference where p > 0.05. Figure S3. The cngc2‐3 mutant showed high extracellular Ca2+‐induced cytosolic calcium ([Ca2+]cyt) elevation in guard cells. The guard cell [Ca2+]cyt elevation of wild‐type (WT) and cngc2‐3 plants expressing Nuclear Export Signal (NES)‐fused Yellow Cameleon3.6 (NES‐YC3.6) was monitored. (A–D) Representative traces of fluorescence emission ratios (F535/F480 [file PPL-177-e70396-s001.pdf]

Supplementary Figures

CNGC2 Negatively Regulates Stomatal Closure and Is not Required for flg22- and H_2O_2-Induced Guard Cell $[Ca^{2+}]_{cyt}$ Elevation in *Arabidopsis thaliana*

Rojina Akter[1], Yasuhiro Inoue[1], Saori Masumoto[2], Yoshiharu Mimata[1], Takakazu Matsuura[3], Izumi C. Mori[3], Toshiyuki Nakamura[1], Yoshimasa Nakamura[1], Yoshiyuki Murata[1], and Shintaro Munemasa[1]*

[1] Graduate School of Environmental and Life Science, Okayama University, Okayama 700-8530, Japan.

[2] Faculty of Agriculture, Okayama University, Okayama 700-8530, Japan.

[3] Institute of Plant Science and Resources, Okayama University, Kurashiki, Okayama, 710-0046, Japan

***Correspondence:** Shintaro Munemasa (smunemasa@okayama-u.ac.jp)

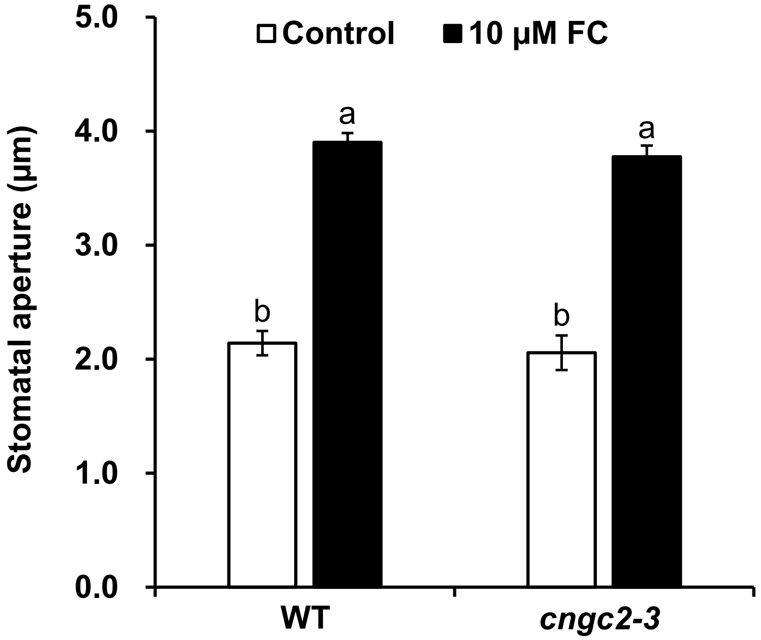

Supplementary Figure S1. Fusicoccin (FC)-mediated stomatal opening in WT and the *cngc2-3* mutant. Averages of stomatal apertures from three independent experiments (n=3, total 60 stomata) are shown. In this figure, Col-0 was used as wild-type (WT). Data are expressed as mean ± SE. Statistical differences were analyzed by one-way ANOVA with Tukey's test. Different letters represent significant differences ($P < 0.05$).

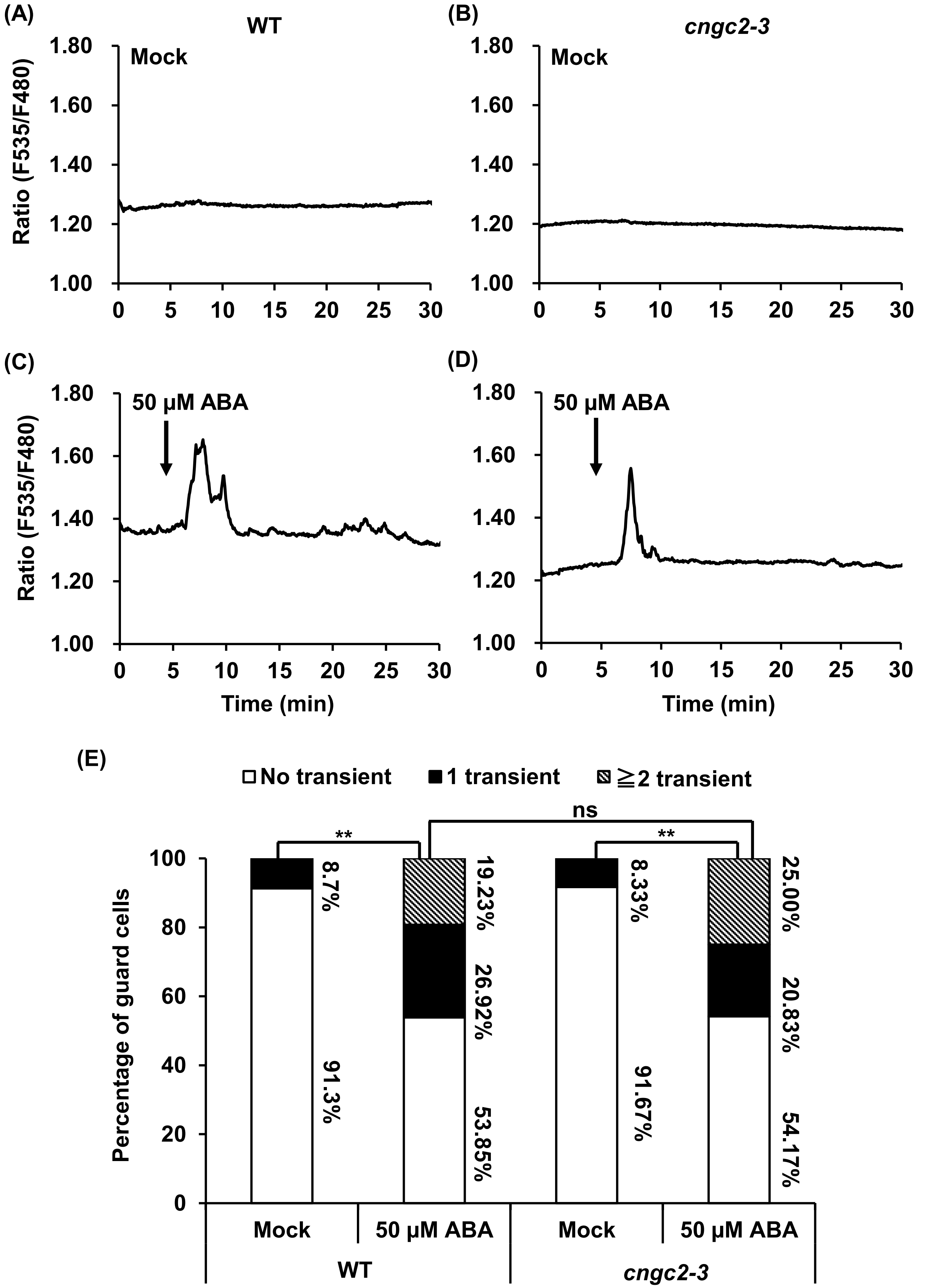
(A)
WT
Mock
Ratio (F535/F480)
1.80
1.60
1.40
1.20
1.00
0 5 10 15 20 25 30
(B)
cngc2-3
Mock
(C)
50 μM ABA
Time (min)
(D)
50 μM ABA
Time (min)
(E)
No transient
1 transient
≧2 transient
ns
**
**
Percentage of guard cells
100
80
60
40
20
0
8.7%
91.3%
19.23%
26.92%
53.85%
8.33%
91.67%
25.00%
20.83%
54.17%
Mock
50 μM ABA
Mock
50 μM ABA
WT
cngc2-3

Supplementary Figure S2. The *cngc2-3* mutant showed ABA-induced cytosolic calcium ($[Ca^{2+}]_{cyt}$) elevation in guard cells. The guard cell $[Ca^{2+}]_{cyt}$ elevation of wild-type (WT) and *cngc2-3* plants expressing Nuclear Export Signal (NES)-fused Yellow Cameleon3.6 (NES-YC3.6) was monitored. (A-D) Representative traces of fluorescence emission ratios (F535/F480) showing ABA-induced $[Ca^{2+}]_{cyt}$ transients in guard cells. In mock treatments, guard cells were incubated in stomatal assay buffer for 2 h in light, and no ABA applied during the measurement of fluorescence emission ratios (A and B). In ABA treatments, 50 μM ABA were applied to the guard cells in stomatal assay buffer 5 min after the measurement (C, D). (E) Percentage bar chart represents the percent (%) of guard cells showing transient $[Ca^{2+}]_{cyt}$ elevations. The number of transient $[Ca^{2+}]_{cyt}$ elevations under mock and 50 μM ABA treatment in WT and *cngc2-3* mutants were counted when the fluorescence ratio (F535/F480) increased ≥0.1 unit from the baseline. In this figure, Col-0 was used as wild-type (WT). The significance of differences between different treatments were determined by chi-squared ($\chi 2$) test, *$P < 0.05$, **$P < 0.01$. The "ns" indicates non-significant difference where $P > 0.05$.

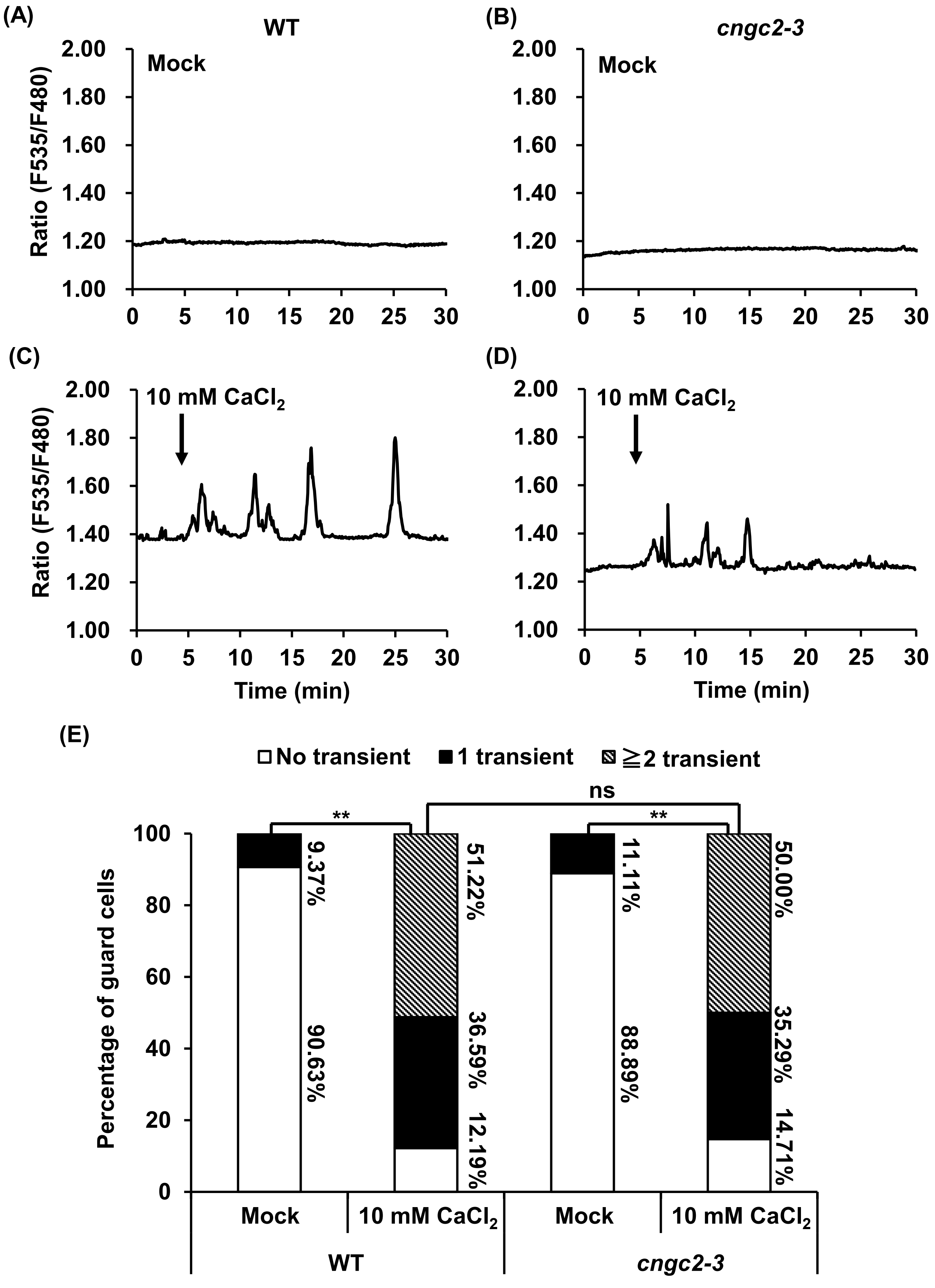

(A)
WT
Mock
Ratio (F535/F480)
2.00
1.80
1.60
1.40
1.20
1.00
0
5
10
15
20
25
30
(B)
cngc2-3
Mock
(C)
10 mM CaCl2
Time (min)
(D)
10 mM CaCl2
Time (min)
(E)
No transient
1 transient
≧2 transient
ns
**
**
Percentage of guard cells
100
80
60
40
20
0
9.37%
90.63%
51.22%
36.59%
12.19%
11.11%
88.89%
50.00%
35.29%
14.71%
Mock
10 mM CaCl2
Mock
10 mM CaCl2
WT
cngc2-3

Supplementary Figure S3. The *cngc2-3* mutant showed high extracellular Ca^{2+}-induced cytosolic calcium ($[Ca^{2+}]_{cyt}$) elevation in guard cells. The guard cell $[Ca^{2+}]_{cyt}$ elevation of wild-type (WT) and *cngc2-3* plants expressing Nuclear Export Signal (NES)-fused Yellow Cameleon3.6 (NES-YC3.6) was monitored. (A-D) Representative traces of fluorescence emission ratios (F535/F480) showing extracellular Ca^{2+}-induced $[Ca^{2+}]_{cyt}$ transients in guard cells. In mock treatments, guard cells were incubated in stomatal assay buffer for 2 h in light, and no extracellular Ca^{2+} applied during the measurement of fluorescence emission ratios (A and B). In extracellular Ca^{2+} treatments, 10 mM $CaCl_2$ were applied to the guard cells in stomatal assay buffer 5 min after the measurement (C, D). (E) Percentage bar chart represents the percent (%) of guard cells showing transient $[Ca^{2+}]_{cyt}$ elevations. The number of transient $[Ca^{2+}]_{cyt}$ elevations under mock and 10 mM $CaCl_2$ treatment in WT and *cngc2-3* mutants were counted when the fluorescence ratio (F535/F480) increased ≥0.1 unit from the baseline. In this figure, Col-0 was used as wild-type (WT). The significance of differences between different treatments were determined by chi-squared (χ2) test, *$P < 0.05$, **$P < 0.01$. The “ns” indicates non-significant difference where $P > 0.05$.
